# Immune Complex-Induced, Nitric Oxide-Mediated Vascular Endothelial Cell Death by Phagocytes Is Prevented with Decoy FcγReceptors

**DOI:** 10.1371/journal.pone.0153620

**Published:** 2016-04-21

**Authors:** Ramanjaneya V. R. Mula, Deepa Machiah, Lauren Holland, Xinyu Wang, Harish Parihar, Avadhesh C. Sharma, Periasamy Selvaraj, Rangaiah Shashidharamurthy

**Affiliations:** 1 Department of Pharmaceutical Sciences, Philadelphia College of Osteopathic Medicine - School of Pharmacy, Suwanee, Georgia, United States of America; 2 Department of Pharmacy Practice, Philadelphia College of Osteopathic Medicine - School of Pharmacy, Suwanee, Georgia, United States of America; 3 Department of Molecular Pathology Laboratory, Yerkes National Primate Research Centre, Atlanta, Georgia, United States of America; 4 Department of Pathology and Laboratory Medicine, Emory University, Atlanta, Georgia, United States of America; National Institutes of Health, UNITED STATES

## Abstract

Autoimmune vasculitis is an endothelial inflammatory disease that results from the deposition of immune-complexes (ICs) in blood vessels. The interaction between Fcgamma receptors (FcγRs) expressed on inflammatory cells with ICs is known to cause blood vessel damage. Hence, blocking the interaction of ICs and inflammatory cells is essential to prevent the IC-mediated blood vessel damage. Thus we tested if uncoupling the interaction of FcγRs and ICs prevents endothelium damage. Herein, we demonstrate that dimeric FcγR-Igs prevented nitric oxide (NO) mediated apoptosis of human umbilical vein endothelial cells (HUVECs) in an *in vitro* vasculitis model. Dimeric FcγR-Igs significantly inhibited the IC-induced upregulation of inducible nitric oxide synthase (iNOS) and nitric oxide (NO) release by murine monocytic cell line. However, FcγR-Igs did not affect the exogenously added NO-induced upregulation of pro-apoptotic genes such as Bax (15 fold), Bak (35 fold), cytochrome-C (11 fold) and caspase-3 (30 fold) in HUVECs. In conclusion, these data suggest that IC-induced NO could be one of the major inflammatory mediator promoting blood vessel inflammation and endothelial cell death during IC-mediated vasculitis which can be effectively blocked by dimeric decoy FcγRs.

## Introduction

The immune system has evolved to defend our body against invading pathogens, however under certain conditions it attacks itself, leading to the development of autoimmune diseases. During the development of autoimmune diseases, autoantibodies bind to the antigens and form immune complexes (ICs). During autoimmune vasculitis, circulating ICs deposit in the vascular endothelial walls leading to an infiltration of inflammatory cells [1, 2] causing weakening and narrowing of the blood vessels. This vascular inflammation results in vital organ damage including heart failure and neurological conditions such as stroke.

ICs deposited on the vascular endothelial wall cause the inflammation through two different pathways: activation of inflammatory cells through the binding of FcγRs and by the initiation of the complement pathway. The requirement of FcγR expressing cells during the pathogenesis of IC-mediated inflammatory vascular damage has been demonstrated in humans as well as gene knockout mice models [3, 4]. The interaction of between ICs and the FcγRs expressed on inflammatory cells is a key event in the development of various IC-mediated diseases including vasculitis [5–9] and leads to the destruction of tissues/cells with IC-deposits through antibody dependent cellular cytotoxicity and phagocytosis [6, 10]. Apart from FcγRs interactions, ICs can also mediate damage through the complement pathway [11, 12]. Activation of complement pathway by ICs results in tissue/cell damage directly or indirectly by attracting inflammatory cells. The direct damage to cells through the complement pathway may not be a major mechanism of pathogenesis in many autoimmune inflammatory disorders because of the complement regulatory mechanism. However, as an indirect mechanism, the chemoattractant nature of complement peptide C5a can attract inflammatory cells in addition to the upregulation of FcγRs on macrophages [11, 13]. Once inflammatory cells are attracted by C5a, ICs can then bind to FcγRs on their surface and trigger effector mechanisms [3, 14]. Thus, the FcγR-expression on inflammatory cells might be an essential player in IC-mediated tissue/cell damage. These studies suggest that ligation of FcγRs with ICs results in the activation of inflammatory cells accumulated at the site of IC deposition in blood vessels. Inflammatory mediators released by activated cells are responsible for the endothelial cell inflammation, injury and subsequent vasculitis.

It has been shown that IC-mediated vascular damage is linked to the release of toxic free radicals by activated inflammatory cells such as neutrophils and macrophages [15–17]. In particular, nitric oxide (NO), a highly reactive free radical, is implicated in many IC-mediated inflammatory autoimmune disorders [18]. The critical enzyme involved in the production of NO is the nitric oxide synthase (NOS) which mediates the conversion of L-arginine to L-citrulline and NO. NOS exist as three isoforms nNOS (neuronal) eNOS (endothelial) and iNOS (inducible). Both, nNOS and eNOS are endogenously expressed and known to be required to maintain normal physiological functions. Whereas many inflammatory disorders are associated with upregulated iNOS [19–21] whose progression was arrested by iNOS blockers [22]. These data suggest that NO produced from over expressed iNOS may be responsible for causing the tissue damage. Interestingly, exogenous NO has both anti- and pro-apoptotic effects on endothelial cells. This differential function of NO is concentration dependent. At a low concentration (picomolar to nanomolar), NO induces survival signals by upregulating anti-apoptotic proteins, whereas a high concentration of NO (micromolar) initiates the pro-apoptotic pathway [23, 24]. These results suggest that the normal physiological levels of NO are protective during any external or internal endothelial damage but will be cytotoxic at higher concentration produced during certain inflammatory conditions. In support of this, it has been shown that murine macrophages produce up to 1mM concentration of NO in an *in vivo* atherosclerosis model suggesting that macrophages might be the major cells to produce high levels of NO *in vivo* [25, 26]. Thus, NO works as a double-edged sword protecting normal physiological functions at a low amount and its higher levels resulting in nitrosative stress induced apoptosis of cells. IC interactions with macrophages or effector cells trigger iNOS upregulation resulting in the production of copious amounts of NO leading to tissue injury [15, 17, 23].

The current study was designed to test the hypothesis that extracellularly secreted NO by IC-activated inflammatory cells is a major factor in causing inflammatory vascular endothelial cell death (vascular damage) during autoimmune vasculitis. Herein we used murine monocytic cell line as model for *in vitro* vasculitis studies. Our data demonstrates that blocking IC binding to FcγRs expressed on inflammatory cells using recombinant dimeric FcγR-Ig molecules is an effective way to inhibit IC-mediated NO production and subsequent vascular damage.

## Materials and Methods

### Cell lines and reagents

Human umbilical vein endothelial cells (HUVECs) and murine monocytic cell line RAW264.7 were purchased from ATCC (Manassas, VA). Medium M200, low serum growth supplement (LSGS), EDTA (0.5% trypsin—0.02% EDTA), penicillin G and streptomycin sulfate, and super script first strand cDNA synthesis kit were purchased from Life Technologies (Grand Island, NY). Gene specific primers were from Real Time Primers, LLC (Elkins Park, PA). Caspase-3 enzyme assay kit from Molecular Probes (Eugene, Oregon). Dimethyl sulfoxide; *Escherichia coli* LPS; Endothelial cell growth supplement (ECGS) and 1400W small molecule specific inhibitor for iNOS and PKH26 red fluorescent cell membrane labelling kit were purchased from Sigma Aldrich (St. Louis, MO). Ovalbumin, rabbit anti-Ovalbumin IgG antibodies, S-Nitroso-N-Acetyl-D, L-Penicillamine (SNAP) were procured from Cayman chemicals (Ann Arbor, Michigan). The CellTiter-Blue Assay kit from Promega (Madison, WI). RIPA lysis buffer and protease inhibitor cocktail were purchased from Santa-Cruz biotechnology (Dallas, Texas). SYBR RT-PCR master mix was purchased from Thermo-Fisher Scientific (Waltham, MA). Fc receptor blocking 2.4G2 monoclonal antibody that react specifically with mouse Fcγ receptors was procured from Tonbo biosciences (San Diego, CA). RNeasy Mini kits were bought from Qiagen (Valencia, CA).

### Cell culture and IC preparation

HUVECs were cultured as described [27]. Briefly, HUVECs were serially propagated in M200 supplemented with LSGS. Early-passage cultures were used in all the experiments. RAW264.7 cells were cultured in RPMI 1640 medium supplemented with 10% heat inactivated fetal bovine serum. Both the cell lines were maintained with 100 U/ml penicillin and 100μg/ml streptomycin and incubated at 37°C in 5% CO_2_ + 95% humidity. IC was prepared as we previously described [28, 29]. Briefly, egg ovalbumin and anti-ovalbumin rabbit IgG were mixed in a ratio of 3:1 (w/w) and incubated overnight at 4°C and the soluble ICs were used for the study. All the stimulation experiments were carried out in serum-free medium unless stated otherwise.

### Expression and purification of recombinant soluble CD16A-Ig and CD32A-Ig

The construction and expression of dimeric form of CD16A-Ig and CD32A-Ig by ligating the extracellular domain of human CD16A or CD32A to Fc domain of the human IgG1 heavy chain has been reported by our group [28, 30]. The recombinant molecules were purified from CHO cell transfectants using a protein-G Gamma Bind Plus (Pharmacia Biotech, NJ) Sepharose column as described [28]. The purified CD16A-Ig and CD32A-Ig molecules were analyzed by 10% SDS-PAGE under reducing and non-reducing conditions and the protein bands were visualized by silver staining and Western blot. Protein concentration was measured using the Micro BCA protein assay kit (Pierce, NJ) with BSA as a standard.

### *In vitro* vasculitis assay

HUVECs (1x10^6^ cells/well) were cultured to confluence in 12 well tissue culture plates as described above. HUVECs secrete fibronectin and the secreted fibronectin binds integrin molecules expressed on HUVECs [31]. We used polyclonal rabbit anti-human fibronectin IgG antibodies to coat the surface of HUVECs. This serves as model for *in vitro* IC deposited vasculitis in our studies and referred as antibody-coated HUVECs throughout the text. Briefly, HUVECs were incubated with rabbit anti-human fibronectin IgG for 2 hr in serum free medium at 4°C. 100μg of dimeric FcγR-Igs (CD16A-Ig and CD32A-Ig) were added to the antibody coated HUVECs and continued incubation for 1hr at 37°C. RAW264.7 cells membrane was labeled with PKH26 red fluorescent dye. PKH26-labeled RAW264.7 cells pre-treated with 2.4G2 were over layered on antibody-coated HUVECs, uncoated HUVECs and uncoated HUVECs over layered with PKH26-labelled RAW264.7 cells served as specificity control. The cells were incubated for 2hr at 37°C. The plate was taken out and inverted slowly into pre-chilled 1xPBS in an ice bucket carefully without disturbing the cells. The unbound RAW264.7 cells came off by gravity and the bound cells were read at 560nm using BioTek (Winooski, VT) plate reader.

### Detection of IC-induced NO and iNOS levels

RAW264.7 cells were incubated at 37°C for 12 and 24hr with various concentrations of the ICs to detect the IC-induced NO production and iNOS upregulation. To study whether FcγR-Ig molecules block the NO secretion and iNOS upregulation, the experiments were carried out in the presence or absence of various concentrations of FcγR-Ig molecules (0–300 μg/ml) at different time points (0–24 hr). Antibody-coated HUVECs, uncoated HUVECs, antigen alone, antibody alone and FcγRs blocking antibody (2.4G2) treated cells were served as specificity controls. Culture supernatant was collected and stored at -80°C for the estimation of IC-induced NO production.

The NO present in biological fluids is recovered in nitrite and nitrate form [19]. The colorimetric Griess reagent system (Promega Corporation, Madison) was used to estimate the concentration of nitrite/nitrate in the samples to assess NO production by the cells [19].

Total protein extracts were prepared using the RIPA lysis buffer and stored at -80°C until used to detect iNOS upregulation by Western blot analysis. The target protein was identified using primary antibodies specific for iNOS, caspase3, α-Tubulin, GAPDH, and β-actin. LiCOR Goat anti-rabbit and Goat anti-mouse secondary antibodies (IRDye800/IRDye680) were used as detection antibodies. The fluorescence in the membranes was scanned using LiCOR Imaging system. Quantitation of the blots was carried out by densitometric analysis using ImageJ software (NIH. Bethesda, MA).

### iNOS Inhibition and cell viability assay

iNOS inhibition assay was carried out as described [32]. Briefly, the antibody-coated HUVECs were co-incubated for 4, 8 and 12 hrs at 37°C with RAW264.7 cells pre-treated with iNOS inhibitor 1400W (100ng/mL, for 1hr) and continuously present throughout the experiment. The plates were taken out at specified time points and RAW264.7 cells over layer were washed carefully without disturbing the HUVECs layer. Cell viability assay was performed using CellTiter-Blue Assay as per manufactures instruction (Promega, Madison, WI) for estimating the number of viable cells. The fluorescent signal was read at 562 nm using BioTek plate reader (Winooski, VT).

### Real-time Polymerase Chain Reaction (RTPCR)

HUVECs treated with different concentrations of SNAP (0-2mM) for 24hr in serum free M200 medium and then washed twice with 1xPBS buffer and total RNA was extracted using RNeasy mini kit (Qiagen). The total RNA (0.5μg) was used for cDNA synthesis. Specific mRNA transcript levels of genes differentially expressing in HUVECs were assessed using an SYBR green-based quantitative PCR method using Bio-Rad iQ5 system. The gene specific sequences of primers used were as follows. Bak1: forward 5'-TTTGCAGTTGGACTCTCAGG -3', reverse 5'-GTAGTCTGGGCAAGGAGGAG-3'; Bax: forward 5'-GCTGGACATTGGACTTCCTC-3', reverse 5'-CTCAGCCCATCTTCTTCCAG-3'; cytochrome-C: forward 5'- GTTGAAAAGGGAGGCAAGCA-3' reverse 5'- TGTTCTTATTGGCGGCTGTG-3'; caspase-3: forward 5'-CCCCTGGATCTACCAGCATA-3', reverse 5'-TGTCTCTGCTCAGGCTCAAA-3' and β-actin; forward 5'-GGACTTCGAAGAGATGG-3', reverse 5'-AGCACTGTGTTGGCGTACAG-3'. Data analysis has shown that the β-actin transcription levels were not affected under the given experimental conditions. The results were analyzed as described [33].

### Caspase 3 enzyme activity assay

HUVECs were incubated with various concentrations of SNAP (0-2mM) in 2ml of serum free M200 medium at different time points (4, 8 and 24hr). The cells were harvested and centrifuged at 5000 rpm for 5min. The culture supernatant was removed, the cell pellet was lysed and caspase-3 enzyme assay was carried out (Molecular Probes, Inc. Eugene, Oregon). The caspase-3 enzymatic activity in terms of fluorescence was measured at 485/520nm. Upregulation of caspase-3 was detected by Western blot analysis using same cell lysate by specific rabbit anti-caspase-3 as a primary antibody and IRDye800 conjugated goat anti-rabbit antibodies as a detection antibody.

### Statistical evaluation

The statistical analysis was performed using SPSS v22 (IBM corp, Chicago, IL, USA). The data were analyzed using Student’s t-test and one way analysis of variance (one way ANOVA) followed by post-hoc least significant difference (LSD) test for intra- and inter-group statistical differences. The protein level and gene expression results were expressed as means ± SEM obtained from three to six independent experiments and p values ≤0.05 was considered as statistically significant and ≤0.005 as highly significant.

## Results

### Antibody-mediated cellular toxicity in HUVECs was blocked by dimeric FcγR-Ig molecules

**Fig 1:**
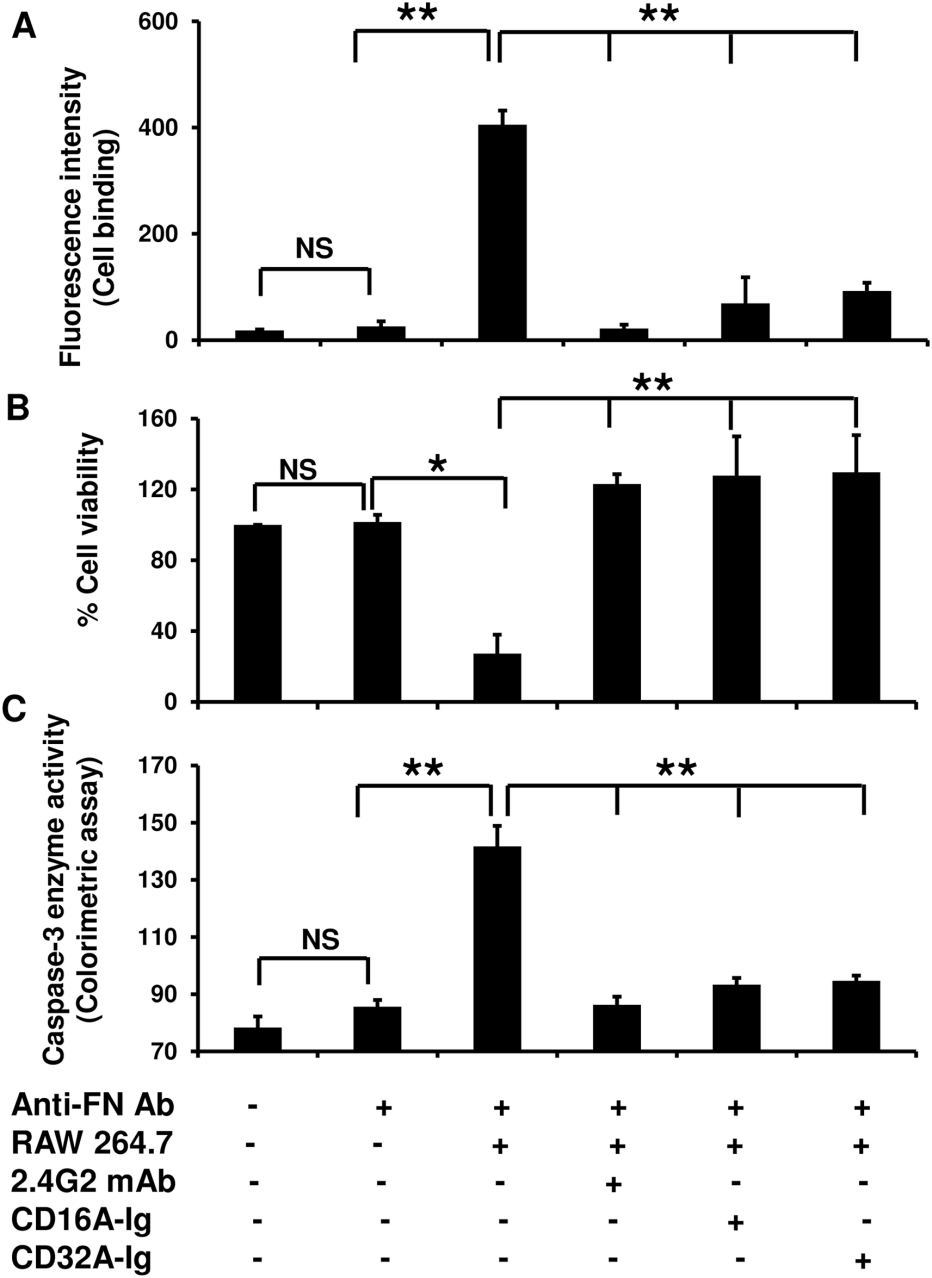
FcγR-Ig blocks the macrophage mediated antibody-coated endothelial cell death. **(A)** RAW 264.7 cells were co-cultured with HUVECs coated with anti-human fibronectin Ab or HUVECs alone in the presence or absence of CD16A-Ig and CD32A-Ig at 37°C. **(A)** The cells were analyzed after 2hr for binding of RAW264.7 cells with HUVECs using plate inversion method. **(B)** After 8hr, the HUVECs viability was analyzed using CellTiter-Blue Assay method. **(C)** After 12hr caspase-3 assay was carried out by calorimetric method. Blocking mAbs (2.4G2), CD16A-Ig, and CD32A-Ig were pre-incubated for 1 hr at 4°C and then continuously present during their incubation at 37°C. In all the experiments RAW 264.7 cells pretreated with 2.4G2 mAb, antigen alone, antibody alone, HUVEC treated with medium alone served as controls. Data shown are the average of three individual experiments; each experiment was carried in triplicate. P value < 0.05 considered as significant (*) and P< .005 as highly significant (**), NS: non-significant.

Previously we showed FcγR-Ig molecules (CD16A-Ig, CD32A-Ig) effectively compete with cell surface FcγRs *in vivo* and block IC-mediated skin inflammation in a mouse model [28, 30]. Therefore, first we investigated whether aforementioned dimeric FcγR-Ig molecules compete with cell surface FcγRs expressed on inflammatory cells such as macrophages *in vitro* and block their binding to antibody coated endothelial cells, which is the hallmark of IC-mediated damage to the endothelial cells in an autoimmune vasculitis. To mimic the IC deposited in endothelial cell wall, the HUVECs were coated with an antibody specific for fibronectin deposited on HUVECs. Both CD16A-Ig and CD32A-Ig significantly (P<0.005) blocked (upto 90%) the binding of mouse monocytic cell line (RAW264.7) to antibody-coated HUVECs (Fig 1A). The HUVECs alone and antibody coated HUVECs pretreated with FcγRs blocking antibody (2.4G2) served as controls. It has been shown that ligation of cell surface FcγRs expressed on inflammatory or effector cells with ICs induces cell death in target tissue/cells [8, 34]. Therefore, we investigated whether RAW264.7 cells induce cell death in antibody coated HUVECs and decoy FcγR-Ig molecules bound to the antibody coated HUVECs rescue them from cell death. RAW264.7 cells were co-incubated with antibody coated HUVECs for 8hr at 37°C. The viability of the HUVECs was determined using the Cell Track Dye method. RAW264.7 cells induced more than 60% of cell death of HUVECs (Fig 1B) and upregulated pro-apoptotic enzyme caspase-3 activity (Fig 1C). Pre-treating antibody coated HUVECs with dimeric FcγR-Ig (as low as 100μg/ml) molecules completely rescued HUVEC death and prevented caspase-3 activation (Fig 1C). Taken together, these studies suggest that the blockade of inflammatory cells interactions with ICs deposited or antibody-coated HUVECs through Fc receptors is a critical factor to prevent endothelial cell death.

### Dimeric FcγRs block the release of IC-induced inflammatory mediators from RAW264.7 cells

Previous experiments demonstrate that the RAW264.7 cells were responsible for inducing apoptosis in HUVECs. It has been shown that the pro-inflammatory cytokine secretion and generation of toxic superoxide radicals by activated inflammatory cells are responsible for inducing cell death in target tissue [35, 36]. Therefore, we investigated whether the RAW264.7 cells activated by ICs *via* Fc receptors release inflammatory mediators such as proinflammatory cytokines and superoxide radicals includes nitric oxide (NO) and we investigated if release can be blocked by dimeric FcγR-Ig molecules. RAW264.7 cells were incubated with different concentrations of *in vitro* prepared soluble ICs at 37°C and culture supernatant was collected at different time points to analyze the secretion of cytokines and NO. IC-induced the secretion of many pro-inflammatory cytokines such as IL-1β, IL-6, TNF-α, and G-CSF in a dose (100 and 200μg/ml) and time (12 and 24hr) dependent fashion (S1 Fig). Interestingly, when co-incubation of antibody coated HUVECs, RAW 264.7 cells released significantly (P = 0.005) high concentration of NO (12 folds) into the culture media (S2 Fig).

**Fig 2:**
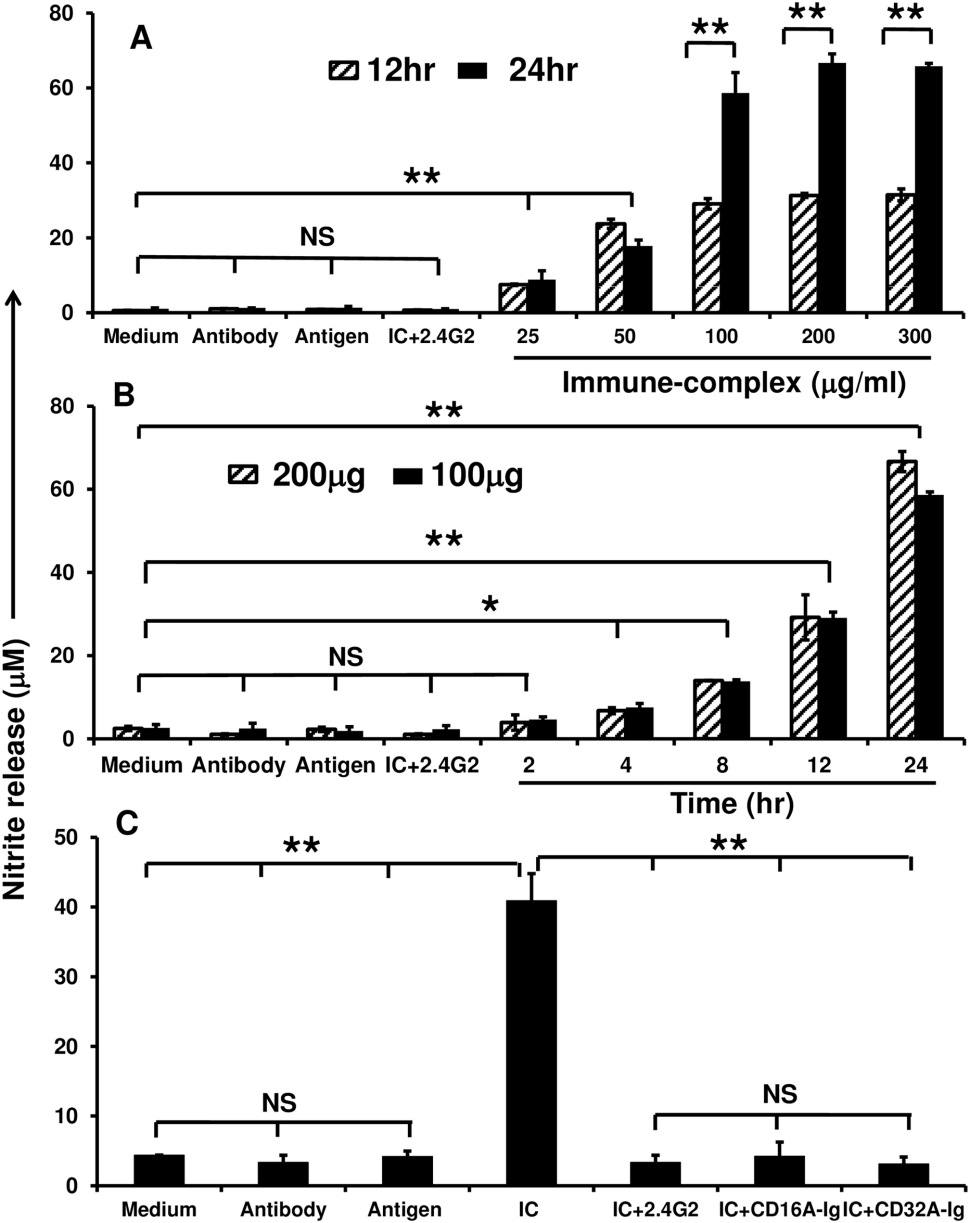
Immune-complex induced nitric oxide secretion is prevented by dimeric FcγR-Ig molecules. **(A)** RAW 264.7 cells were cultured with different concentrations (0–300μg/ml) of soluble ICs in serum free RPMI1640 medium for 12 and 24 hrs. Untreated RAW264.7 cells and cells treated with antigen, antibody and 2.4G2 mAb served as specificity controls. The culture medium was collected at specified time points, and the nitrite concentration was determined using the Griess reagent. **(B)** RAW 267.4 cells were cultured with 100 and 200μg/ml of soluble ICs for different time points (0–24 hr). The culture medium was collected at specified time points, and the nitrite concentration was determined using the Griess reagent. **(C)** CD16A-Ig and CD32A-Ig blocked the binding of ICs to RAW 264.7 cells thereby inhibited the NO production. In all the experiments RAW 264.7 cells pretreated with 2.4G2 mAb, antigen alone, antibody alone, cells treated with medium alone served as specificity controls. Data shown are the average of three individual experiments; each experiment was carried in triplicate. P < 0.05 considered as significant (*) and P< 0.005 as highly significant (**), NS: non-significant.

To confirm whether the secreted NO is induced by ICs *via* FcγRs activation in RAW264.7 cells, the cells were incubated with different concentrations of *in vitro* prepared soluble ICs (0–300μg/ml) and supernatants were harvested at different time intervals (12 and 24hr). NO released into the medium by IC-activated RAW264.7 cells reached the saturation within 24 hr and a minimum of 100μg/ml of IC was enough to induce high level secretion of NO (more than 60μM) into the media when compared to unstimulated cells (Fig 2A and 2B). Interestingly, as low as 100μg/ml of FcγR-Ig molecules completely blocked (around 90%) the release of IC-induced NO by RAW264.7 cells (Fig 2C). In addition, the RAW264.7 cells pre-treated with 2.4G2 mAb completely blocked the NO release. Cells treated with medium, anti-ovalbumin antibody (200μg/ml) and ovalbumin antigen (200μg/ml) alone did not induce NO secretion and served as specificity controls. Taken together, these results suggest that activation of FcγRs by ICs is solely responsible for the induction of above mentioned proinflammatory cytokines and NO in our experimental model and can be effectively blocked by dimeric decoy FcγR-Ig molecules.

### IC-induced iNOS upregulation was inhibited by decoy FcγR-Ig molecules

**Fig 3:**
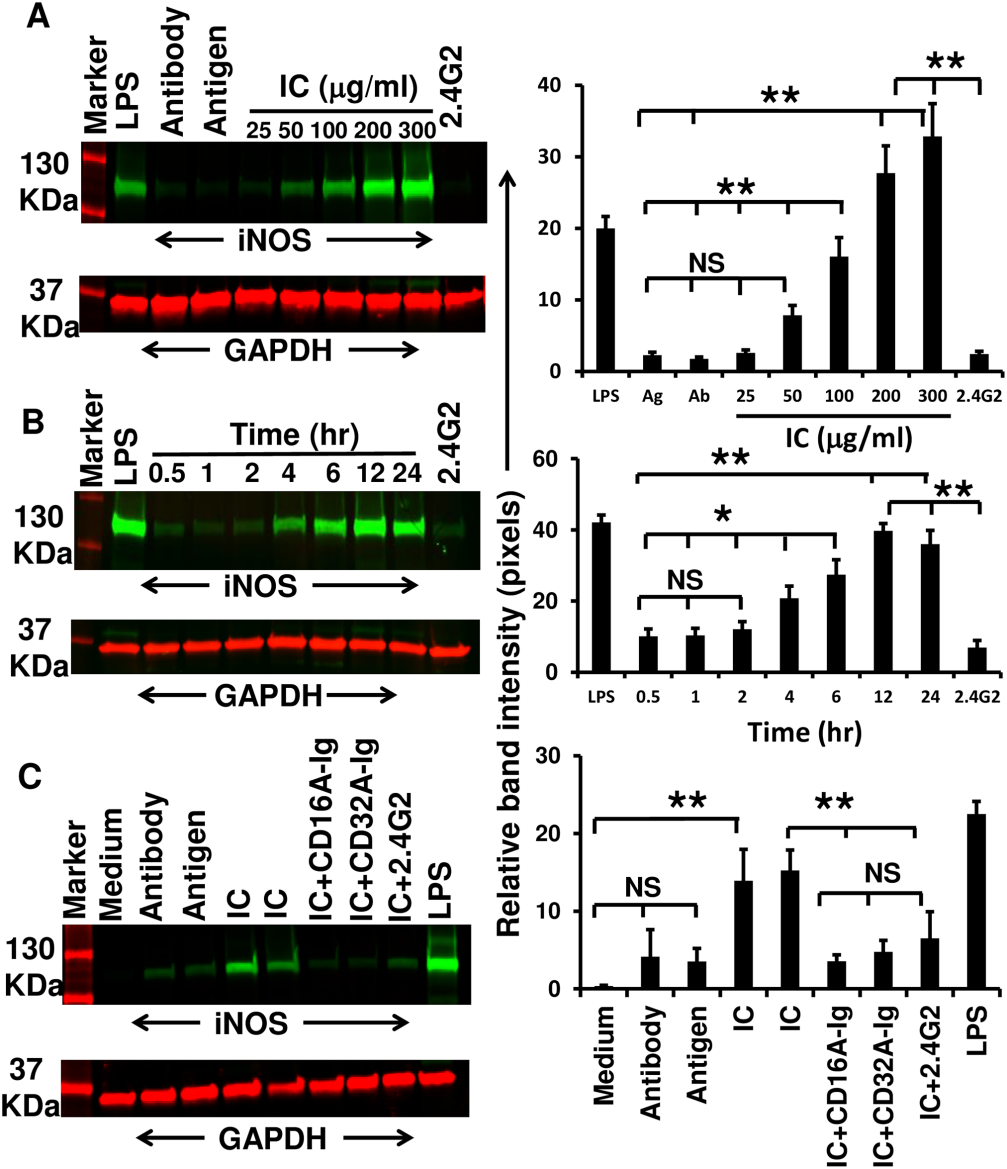
Immune-complex induced iNOS upregulation inhibited by decoy FcγR-Igs. **(A)** RAW 264.7 cells (2×10^6^ cells/ml) were treated with the indicated concentrations of immune complex (25–300μg/ml) in serum free RPMI 1640 medium for 24 hr. **(B)** RAW264.7 cells were treated with 200μg/ml of ICs for different time points (0.5- 24hr). **(C)** CD16A-Ig and CD32A-Ig prevented iNOS upregulation in RAW264.7 cells. RAW264.7 cells (2×10^6^/ml) in serum free medium were blocked from binding to ICs (200μg/ml) by incubating in the presence or absence of CD16A-Ig and CD32A-Ig (100μg/ml) for 24hr at 37°C. Untreated RAW264.7 cells, LPS treated, antigen alone, antibody alone and 2.4G2 mAb treated cells served as specificity controls. Cell lysate was used to detect the iNOS upregulation by Western blot analysis GAPDH was used as internal control. The blots were developed and scanned as described in detail in Materials and Methods. Protein band intensities were analyzed using ImageJ software (NIH, Bethesda, MA) and the relative band intensities were presented. Western blot pictures are representative of three individual experiments. Bar graphs are average of three individual experiments. P< 0.05 considered as significant (*) and < 0.005 as highly significant (**), NS: non-significant.

Previous experiments demonstrated that IC-induced activation of RAW264.7 cells resulted in the secretion of significant (p<0.005) concentrations of NO. As previously reported [22], upregulation of iNOS in activated inflammatory cells is responsible for the release of high concentrations NO [16]. To further substantiate whether the high levels of NO secreted by RAW264.7 cells is mediated by iNOS, we incubated the RAW264.7 cells with various concentrations of soluble ICs (0–300μg/ml) and cell pellets were collected after 24hr. iNOS levels upregulated up to 40 fold as the concentration of IC increased (Fig 3A). However, the difference observed between 200 and 300μg/ml was not statistically significant, suggesting that IC-induced iNOS levels in RAW264.7 cells is dose dependent and reached saturation by 200μg/ml of ICs. Next, we observed time dependent upregulation of IC (200μg/ml) induced iNOS (Fig 3B). Its upregulation initiates after 4hr of incubation and reaches the maximum in 12hr suggesting the time dependent enhanced induction of iNOS by ICs, and the difference observed between 12 and 24hr is not statistically significant (Fig 3B). However, the iNOS upregulation was delayed until 6hr after incubating with 100μg/ml of IC (S3 Fig). The reason for the delayed iNOS upregulation at lower concentration of IC is not clear at this time; however, the concentration of IC may be a critical factor in regulating the iNOS during IC-mediate inflammatory disorders.

Interestingly, dimeric decoy FcγR-Ig molecules efficiently inhibited (up to 90%) the IC-induced iNOS upregulation in RAW264.7 cells, which is comparable with the results observed from 2.4G2 mAb blocking of RAW264.7 cells (Fig 3C). Untreated cells, antigen alone, antibody alone treated cells served as specificity controls as we did not observe iNOS upregulation (Fig 3C). Cells treated with IC alone or LPS served as positive controls. In support of this, the amount of nitrite released into the culture medium by IC-activated RAW264.7 cells (S4 Fig) is highly correlated with iNOS upregulation observed in this experiment (Fig 3B and S3 Fig). Though the iNOS expression reached saturation by 12hr at 200μg/ml of IC (Fig 3B), the amount of NO released is 50% less at 12hr time point compared to 24hr (Fig 2B). This could be due to the cumulative accumulation of nitrates in the culture supernatant over 24hr. These results suggest that IC-induced iNOS upregulation is through activation of FcγRs and can be effectively blocked by decoy FcγR-Ig molecule thereby preventing NO-mediated vascular damage during IC-mediated autoimmune vasculitis.

**Fig 4:**
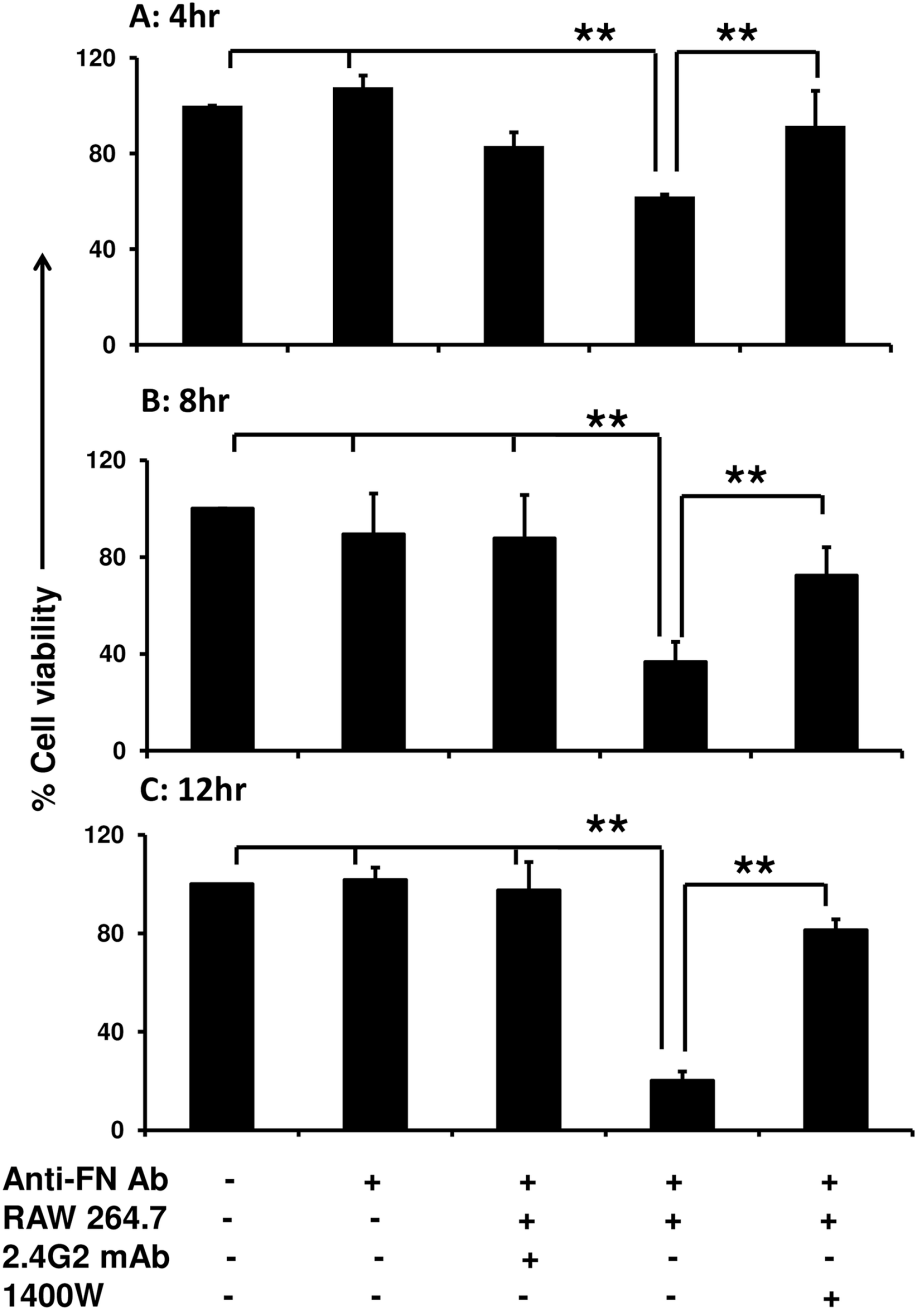
Inhibition of iNOS in RAW264.7 cell prevents NO release and HUVEC death. The RAW264.7 cells were pre-treated with 1400W (100ng/ml) 1hr at 37°C. The HUVECs and pre-treated RAW264.7 cells were co-cultured for 4 **(A)** 8 **(B)** and 12hrs **(C)**. The viability of the HUVECs was determined using CellTitre Blue kit method. RAW264.7 cells pre-treated with 2.4G2 were over layered on antibody-coated HUVECs served as specificity control. Uncoated HUVECs, antibody-coated HUVECs and uncoated HUVECs over layered with 2.4G2 pre-treated RAW264.7 cells were served as additional controls. Data shown are the average of three individual experiments; each experiment was carried in triplicate. P<0.05 considered as significant (*) and P<0.005 as highly significant (**), NS: non-significant.

To further demonstrate that the HUVEC death is specifically induced by NO secreted from macrophages, RAW 276.4 cells were pretreated with inhibitor specific for iNOS (1400W) and then cell viability was performed as mentioned above. We observed that as low as 8hr interaction between antibody-coated HUVEC and macrophage is enough to induce more than 70% cell death in HUVEC (Fig 4B), however, there is no significant increase in cell death after 12hr (Fig 4C). In addition, the macrophages pretreated with 1400W, an iNOS specific inhibitor, completely rescued the HUVEC cell death at the tested time points (Fig 4A–4C) suggesting that iNOS mediated NO secreted from mouse monocytic cells may be a major factor responsible for HUVECs death.

### Exogenously administered NO triggers intrinsic apoptotic pathway in HUVECs, which was not blocked by FcγR-Igs

**Fig 5:**
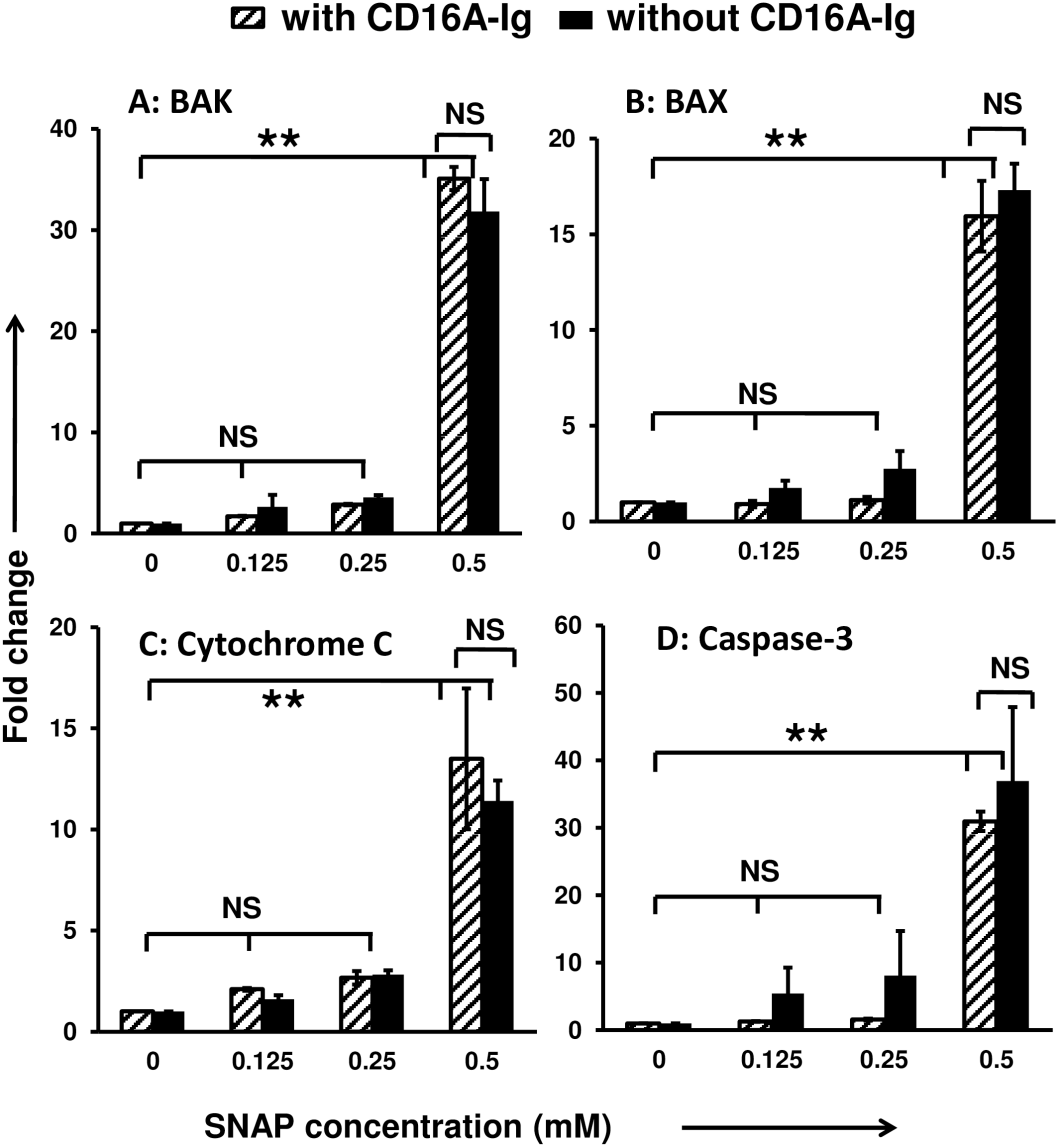
Exogenous nitric oxide initiates intrinsic apoptotic pathway in HUVECs, which was unaltered by decoy FcγR-Igs. Concentration-dependent apoptosis was induced by nitric oxide in cultured HUVECs (1x 10^6^ cells) stimulated with different concentrations of *S*-nitroso-N-acetyl-penicillamine (SNAP) (0–0.5mM) for 24 hr with or without CD16A-Ig was analyzed at mRNA level. Total RNA extracted from the SNAP treated cells was used to make cDNA. QRTPCR analysis of apoptosis related genes **(A)** Bak **(B)** Bax **(C)** Cytochrome-C and **(D)** Caspase-3 were carried out. Data shown are the average of six individual experiments; each experiment was carried in triplicate. P<0.01 were considered as significant (*) and P<0.005 were as highly significant (**), NS: non-significant.

The above mentioned results suggest that the IC-mediated sustained production of NO through iNOS by RAW264.7 cells could be a trigger for the induction of apoptosis in antibody-coated HUVECs. To further substantiate our observations, exogenous NO donor (SNAP) was used to investigate the role of NO in induction of HUVECs apoptosis. The HUVECs were treated with different concentrations (0–0.5 mM) of SNAP for 24 hr. The cDNA was then analyzed for the differential expression of pro-apoptotic genes (Fig 5). It has been shown that, by 24hr 0.5mM of SNAP produces around 100μM of nitrite in the culture medium [37], which is very close to the NO secreted by RAW264.7 cells in our experiments (Fig 2). Exogenously administered NO significantly upregulated the pro-apoptotic genes such as BAK (35fold), BAX (15 fold), cytochrome-C (12 fold), and caspase-3 (30 fold) when compared to untreated cells (Fig 5A–5D). The apoptotic initiation gene cytochrome-C and end point gene caspase-3 have shown a significant (p<0.005) increase in their upregulation in a concentration dependent manner with respect to the exogenously administered NO (Fig 5C and 5D). Co-incubation of FcγR-Ig molecules did not alter the induction of abovementioned pro-apoptotic molecules in antibody-coated HUVECs by exogenous NO administration (Fig 5). These results support that the apoptosis cascade induced in HUVECs is through exogenous NO, but not by the ligation of FcγR-Ig molecules on antibody-coated HUVECs.

**Fig 6:**
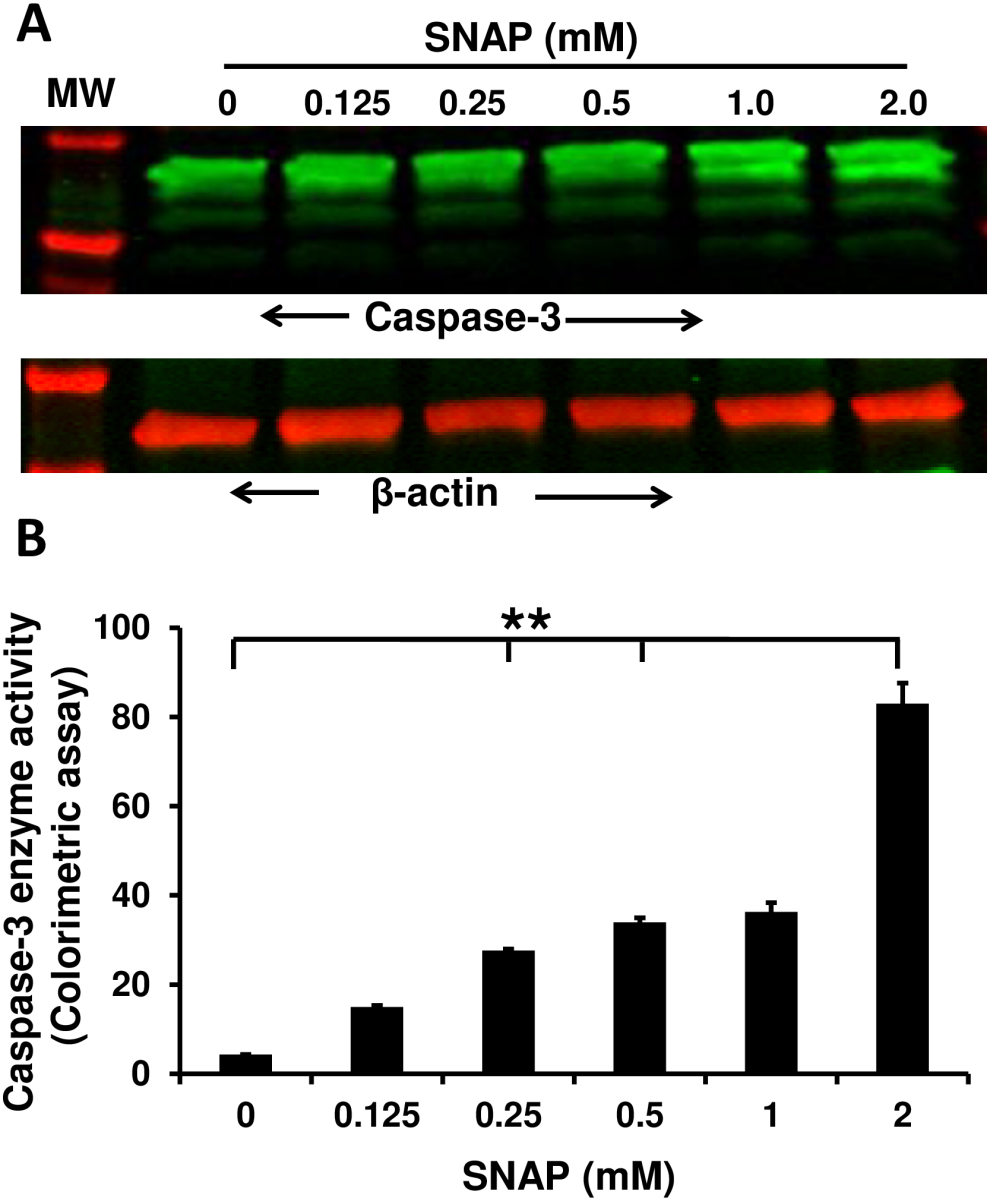
Exogenous nitric oxide induces caspase-3 activation and upregulation in HUVECs. **(A)** Cells treated with different concentrations of *S*-nitroso-N-acetyl-penicillamine (SNAP) (0- 2mM) for 24hr. Untreated HUVECs served as specificity control. Cells were lysed and total proteins extracted. Western blot was carried out using an antibodies specific for caspase-3 and β-actin (internal control). **(B)** Caspase-3 enzymatic activity was performed using total protein extracts. Western blot pictures are representative of three individual experiments. Caspase-3 enzyme activity graph s is average of three individual experiments. P<0.05 considered as significant (*) and P<0.005 as highly significant (**), NS: non-significant.

Irrespective of origin of apoptosis (extrinsic or intrinsic), the end point enzyme (executioner caspase) that triggers the programmed cell death is caspase-3 [38, 39]. Therefore, we have analyzed for caspase-3 activity. The HUVECs were treated with different concentrations of SNAP (NO donor) as described above and harvested after 24hr. Western blot analysis revealed that caspase-3 upregulated in NO-treated HUVECs in a dose dependent manner (Fig 6A) and is consistent with its upregulation at transcriptional level (Fig 5D). In addition, we have also observed the increased enzymatic activity of caspase-3 by colorimetric assay with maximum at 2mM of SNAP after 24 hr of treatment (Fig 6B). Taken together our findings suggest that the NO released either by exogenous NO by SNAP or by IC-activated RAW264.7 cells initiates the intrinsic apoptotic pathway in HUVECs.

## Discussion

During autoimmune vasculitis, the interaction of ICs with FcγRs expressed on inflammatory cells is the key event in initiation of IC-mediated vascular damage [8, 9, 40, 41]. In the recent past, the significance of FcγRs in autoimmune/inflammatory disease conditions has been well-documented [3–7, 14]. The inflammatory mediators such as cytokines and superoxide radicals released from the activated inflammatory cells implicated in the IC-mediated endothelium damage leading to many pathological manifestation including heart failure and stroke [42–46]. However, the mechanisms associated with cellular inflammatory responses and pathways associated with apoptosis of endothelial cells during IC-mediated vasculitis are still not well understood. In the present study, we demonstrated that along with pro-inflammatory cytokines, high levels of NO released from the IC-activated inflammatory cells may directly involve in initiating the programmed cell death in endothelial cells. Upon interaction of ICs, RAW264.7 cells secreted significantly high levels of proinflammatory cytokines such as IL-1β, TNF-α, IL-6, and G-CSF (S1 Fig). It has been shown that these cytokines are directly involved in attracting the inflammatory cells to the site where ICs deposit and also stimulate the endothelial cells to express the adhesion molecules for maximal interaction of inflammatory cells with endothelium [43–46]. Further, recent reports have shown that the increased NO concentration due to over expressed iNOS at local inflammatory microenvironment resulted in higher apoptotic cells in various inflammatory models [47–49]. In addition, Mazaffarian et al., [50] have shown that J774.16 macrophages resulted in an enhanced NO production through cross-linking of Fc receptors with ICs. This is further supported by the upregulation of iNOS by activated macrophages in brain tissues of patients with multiple sclerosis [51] and elevated NO in cerebral cortex and spinal cord during experimental allergic encephalomyelitic conditions [52].

It is well established that physiological levels of NO protect the vasculature from injury whereas excessive NO may be harmful [53]. However, the negative effects of NO secreted by activated inflammatory cells at the microenvironment of IC-mediated endothelial cells damage have not been clearly understood. In the present study, we demonstrated that NO secreted by RAW264.7 cells is through the activation of FcγRs and may directly influence the programmed cell death of endothelial cells. This study also provides the first evidence that extracellularly secreted NO from macrophages could be a possible trigger of pro-apoptotic pathways that accelerate endothelial cell damage. Interestingly, upregulation of iNOS and the NO production could be effectively blocked by decoy FcγR-Ig molecules. These results suggest that the interaction of FcγRs expressed on inflammatory cells with ICs deposited in blood capillaries of various organs results in the initiation of FcγRs activation. The activated FcγRs directly stimulate the upregulation of iNOS, resulting in high level of NO production. The released NO in the microenvironment initiates the pro-apoptotic pathway through cellular oxidative stress leading to target cell/tissue damage.

**Fig 7:**
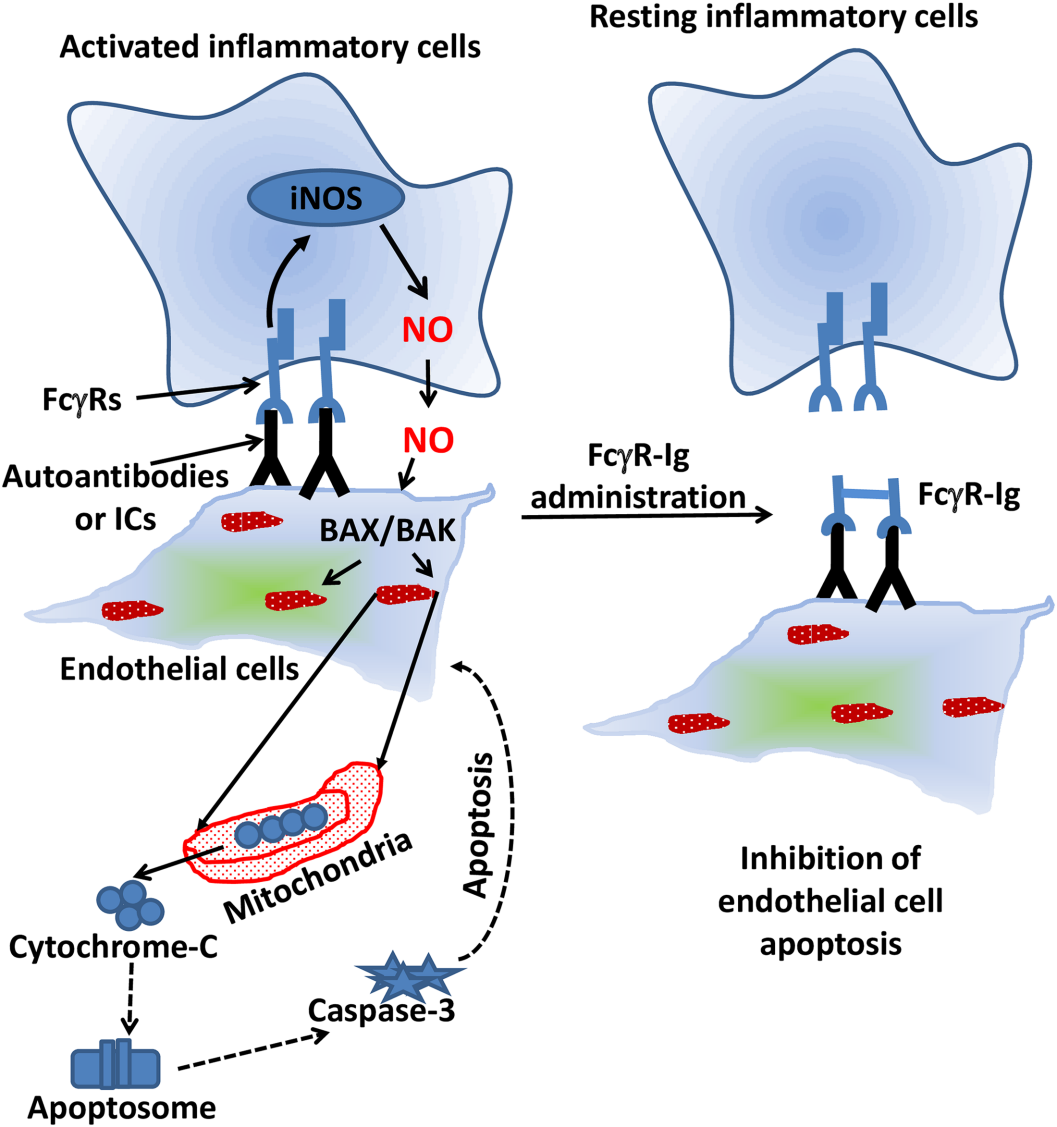
Hypothetical model of inhibition of IC-mediated, NO-induced apoptotic pathway in HUVECs by decoy FcγR-Igs. Circulating ICs deposits in the blood vessels during IC-mediated inflammatory disorders. Effector cells upon binding to the IC get activated. These cells result in the secretion of pro-inflammatory factors like cytokines and toxic superoxide radicals such as nitric oxide in copious amounts. Nitric oxide ability to interact with cellular components in the endothelium results in triggering apoptotic signaling pathway and subsequently cell death and tissue damage. Decoy FcγR-Igs competes with cell surface FcγRs expressed on effector cells and block the access to the ICs deposits in various organs including blood vessels. This will eventually inhibit effector cell mediated endothelial inflammation and damage observed in autoimmune vasculitis.

To further substantiate our findings that NO induces cell death in endothelial cells, we used the SNAP which spontaneously releases NO into the culture medium. We observed significant increase in pro-apoptotic molecules triggered by NO in SNAP treated HUVECs. We analyzed the differential expression of pro-apoptotic genes such as cytochrome-C, caspase-3, BAK and BAX (Fig 5). The results have shown the upregulation of BAX/BAK and cytochrome-C is the indication of intrinsic pathway being triggered to drive the endothelial cells to programmed cell death. Also, the apoptotic end point gene caspase-3 has shown significant changes in its expression at transcriptional level and activation (Figs 5 and 6). In support of this, it has been shown that NO induces the upregulation and translocation of pro-apoptotic Bcl-2 family molecules such as BAX/BAK to mitochondria [54]. In contrast, it has also been suggested that NO can trigger apoptosis by inducing mitochondrial membrane permeabilization with a direct effect on the permeability transition pore complex (PTPC) and/or indirectly by NO-mediated inhibition of oxidative phosphorylation. However, these effects on mitochondria are probably broader and go beyond PTPC opening [55, 56] and this mechanism is excluded because of the presence of endogenous NO and do not correlate with other responses such as caspase activation [39]. These finding are further substantiated by the elevation of caspase-3 activity in antibody-coated HUVECs when co-cultured with RAW264.7 cells (Fig 1). Our data supports that the extracellularly secreted NO upregulates the pro-apoptotic molecules such as BAX/BAK in HUVECs to initiates the intrinsic apoptotic pathway. Possibly the upregulated BAX/BAK facilitates the release of cytochrome-C into the cytosol by inducing mitochondrial permeability. Then, cytochrome-C initiates the series of caspases activation and ultimately caspase-3, which is the point of no return in the process of programmed cell death. The proposed mechanism of action of exogenously secreted NO through activated FcγRs by ICs and the subsequent consequences of endothelial cell death mediated by secreted NO, the significances of blockade of ICs with cell surface FcγRs are depicted in Fig 7.

In conclusion, though the complexity of NO induced cell death signaling exists due to its differential (dichotomous) cellular activities, it is absolutely toxic for cell survival at sustained and chronic level of production as possibly occurring in IC-mediated autoimmune vasculitis. The observations presented here are suggesting the pro-apoptotic function of NO during autoimmune vasculitis. The decoy FcγR-Ig dimeric molecules inhibit the endothelial cell damage by blocking the interactions of ICs with cell surface FcγRs, resulting in inhibiting the toxic oxygen products such as NO by inhibiting iNOS upregulation. Therefore, uncoupling the interaction of ICs with cell surface FcγRs expressed on effector cells could be a novel approach to prevent blood vessel damage during autoimmune vasculitis. Further studies are warranted to understand the cellular complexity with respect to endothelial cell specific determinants of NO signaling in deciding the cell survival or death.

## Supporting Information

S1 Fig Immune-complex induced upregulation of inflammatory cytokines in RAW264.7 cells. Murine monocytic cells were incubated with different concentrations (100, and 200μg/ml) of soluble immune complexes in serum free RPMI1640 medium for 24hr at 37°C. Untreated RAW264.7 cells served as specificity control. The culture medium was collected at specified time points (12 and 24hr). The inflammatory cytokines released into the culture medium were analyzed using and mouse cytokine multi-analyte kit. The inflammatory cytokine data presented here depicts **(A)** TNF-α **(B)** IL-1β **(C)** IL-6 and **(D)** G-CSF. Data are representative of two individual experiments.
(TIF)

S2 Fig RAW264.7 cells secrete high levels of NO upon interaction with antibody-coated HUVECs. HUVECs coated with fibronectin antibody were co-cultured with RAW64.7 cells for 24 hr. The culture medium was collected at specified time point, and the nitrite concentration was determined by using the Griess reagent. Untreated HUVECs alone and uncoated HUVECs co-cultured with RAW264.7 cells, HUVECs coated with anti-fibronectin antibody served as specificity control. The concentration of nitrite released into the medium at 24 hr time point of incubation period was determined by measuring absorbance at 540 nM and comparing it against a standard curve generated using known concentrations of nitrite. Data are average of three independent experiments. P<0.05 considered as significant (*) and P<0.005 as highly significant (**), NS: non-significant.
(TIF)

S3 Fig Immune-complex induced iNOS upregulation inhibited by decoy FcγR-Igs. **(A)** RAW 264.7 cells (2×10^6^ cells/ml) were treated with 100μg/ml of immune-complex for different time points (0–24hr). Cells treated with Lipopolysaccharide and 2.4G2mAb served as specificity controls. iNOS upregulation was analyzed by Western blotting. The membrane was probed with antibodies directed against the rabbit anti-mouse iNOS antibodies and mouse anti-GAPDH antibodies. The blot was developed using the IRDye680/800 conjugated goat anti mouse and goat anti-rabbit secondary antibodies. **(B)** Protein band intensities were analyzed using ImageJ software and relative band intensities were expressed. Photographs are representative of the three individual experiments. Bar graphs are average of three individual experiments.
(TIF)

S4 Fig Time dependent iNOS expression correlates with NO production in mouse monocytic cells. RAW 267.4 cells were cultured with 100 and 200μg/ml of soluble ICs for different time points (0–24 hr). The culture supernatant was collected at different time points, and the nitrite concentration was determined using the Griess reagent. The cells were harvested at the same time point and lysate was analyzed for iNOS expression (Fig 3 and S3 Fig). Data are average of three independent experiments. P<0.05 considered as significant (*) and P<0.005 as highly significant (**), NS: non-significant.
(TIF)
