# Supplementary figures and images for: Immune Complex-Induced, Nitric Oxide-Mediated Vascular Endothelial Cell Death by Phagocytes Is Prevented with Decoy FcγReceptors
Source: PLoS One. 2016 Apr 21;11(4):e0153620. doi: 10.1371/journal.pone.0153620 (PMC4839578; doi:10.1371/journal.pone.0153620)

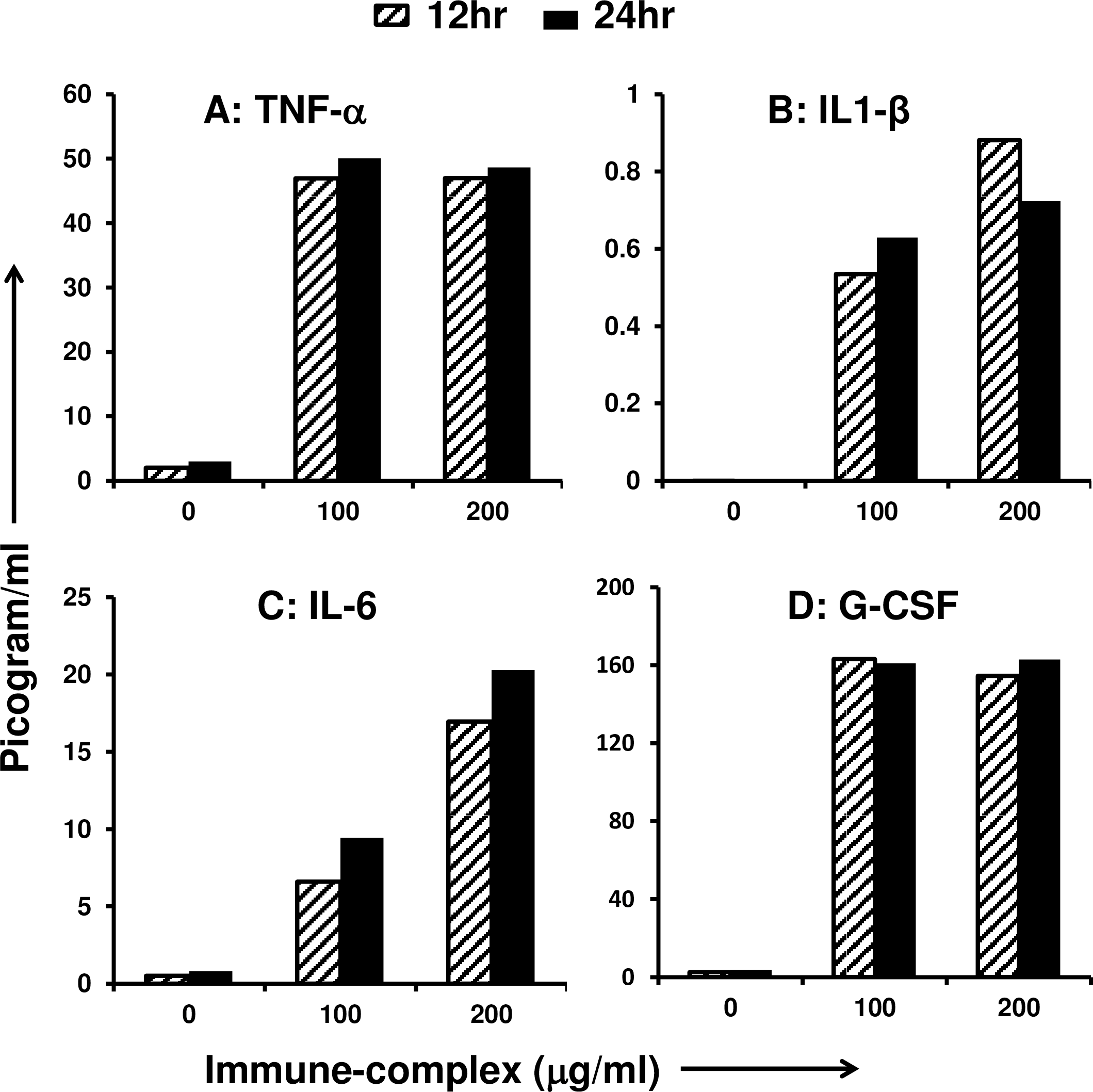

Supplement: S1 Fig — Murine monocytic cells were incubated with different concentrations (100, and 200μg/ml) of soluble immune complexes in serum free RPMI1640 medium for 24hr at 37°C. Untreated RAW264.7 cells served as specificity control. The culture medium was collected at specified time points (12 and 24hr). The inflammatory cytokines released into the culture medium were analyzed using and mouse cytokine multi-analyte kit. The inflammatory cytokine data presented here depicts (A) TNF-α (B) IL-1β (C) IL-6 and (D) G-CSF. Data are representative of two individual experiments. (TIF) [file pone.0153620.s001.tif]

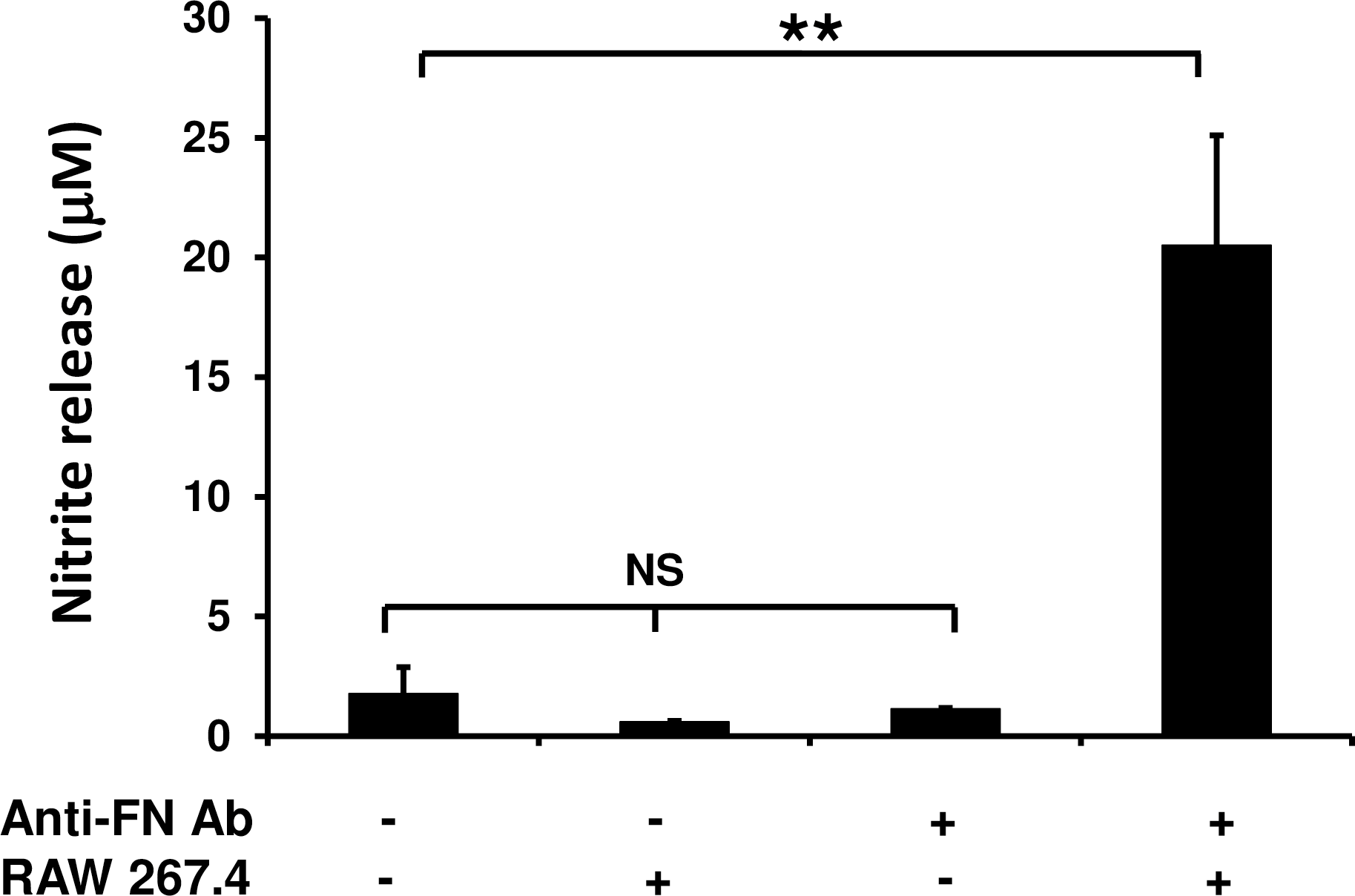

Supplement: S2 Fig — HUVECs coated with fibronectin antibody were co-cultured with RAW64.7 cells for 24 hr. The culture medium was collected at specified time point, and the nitrite concentration was determined by using the Griess reagent. Untreated HUVECs alone and uncoated HUVECs co-cultured with RAW264.7 cells, HUVECs coated with anti-fibronectin antibody served as specificity control. The concentration of nitrite released into the medium at 24 hr time point of incubation period was determined by measuring absorbance at 540 nM and comparing it against a standard curve generated using known concentrations of nitrite. Data are average of three independent experiments. P<0.05 considered as significant (*) and P<0.005 as highly significant (**), NS: non-significant. (TIF) [file pone.0153620.s002.tif]

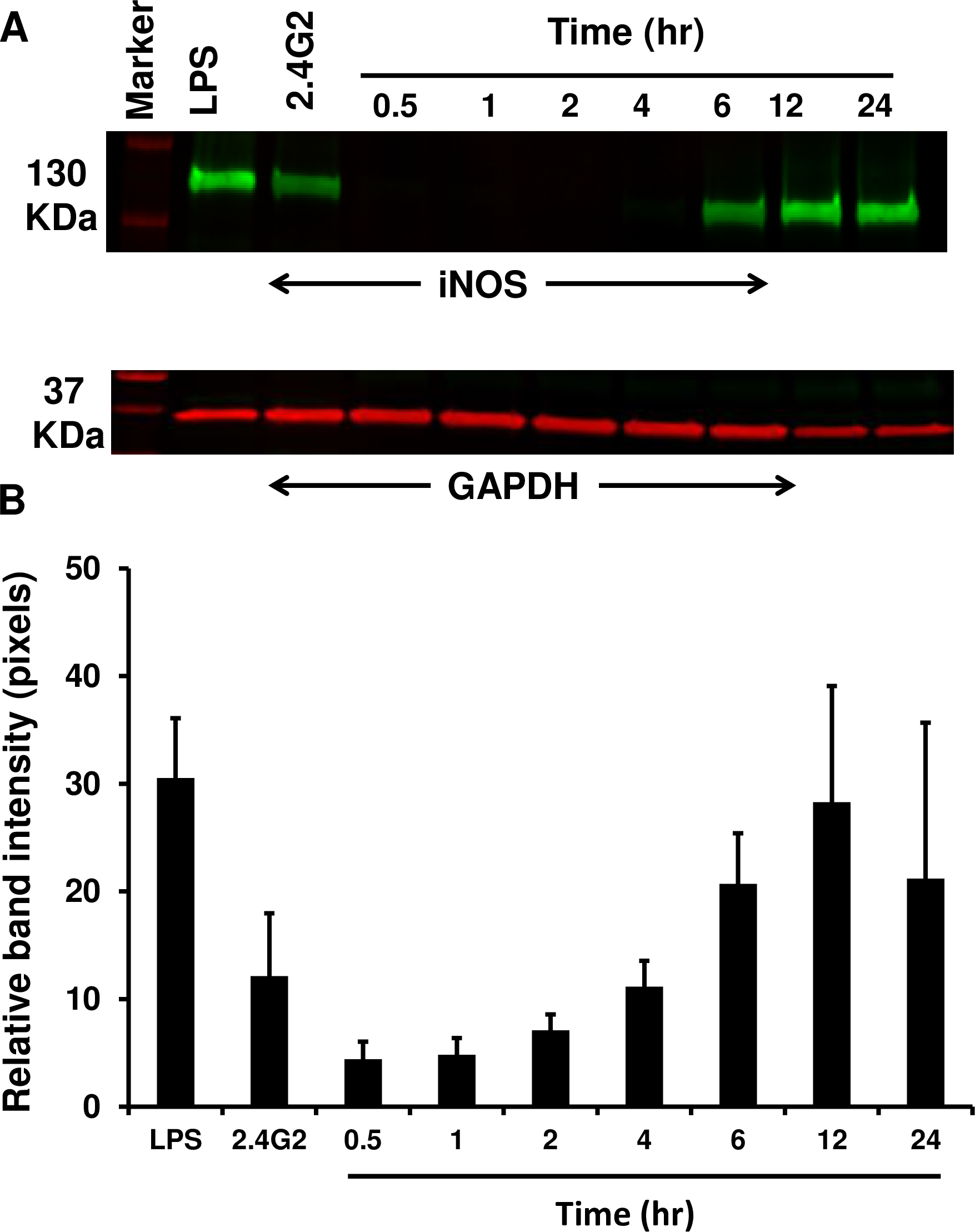

Supplement: S3 Fig — (A) RAW 264.7 cells (2×10^6^ cells/ml) were treated with 100μg/ml of immune-complex for different time points (0–24hr). Cells treated with Lipopolysaccharide and 2.4G2mAb served as specificity controls. iNOS upregulation was analyzed by Western blotting. The membrane was probed with antibodies directed against the rabbit anti-mouse iNOS antibodies and mouse anti-GAPDH antibodies. The blot was developed using the IRDye680/800 conjugated goat anti mouse and goat anti-rabbit secondary antibodies. (B) Protein band intensities were analyzed using ImageJ software and relative band intensities were expressed. Photographs are representative of the three individual experiments. Bar graphs are average of three individual experiments. (TIF) [file pone.0153620.s003.tif]

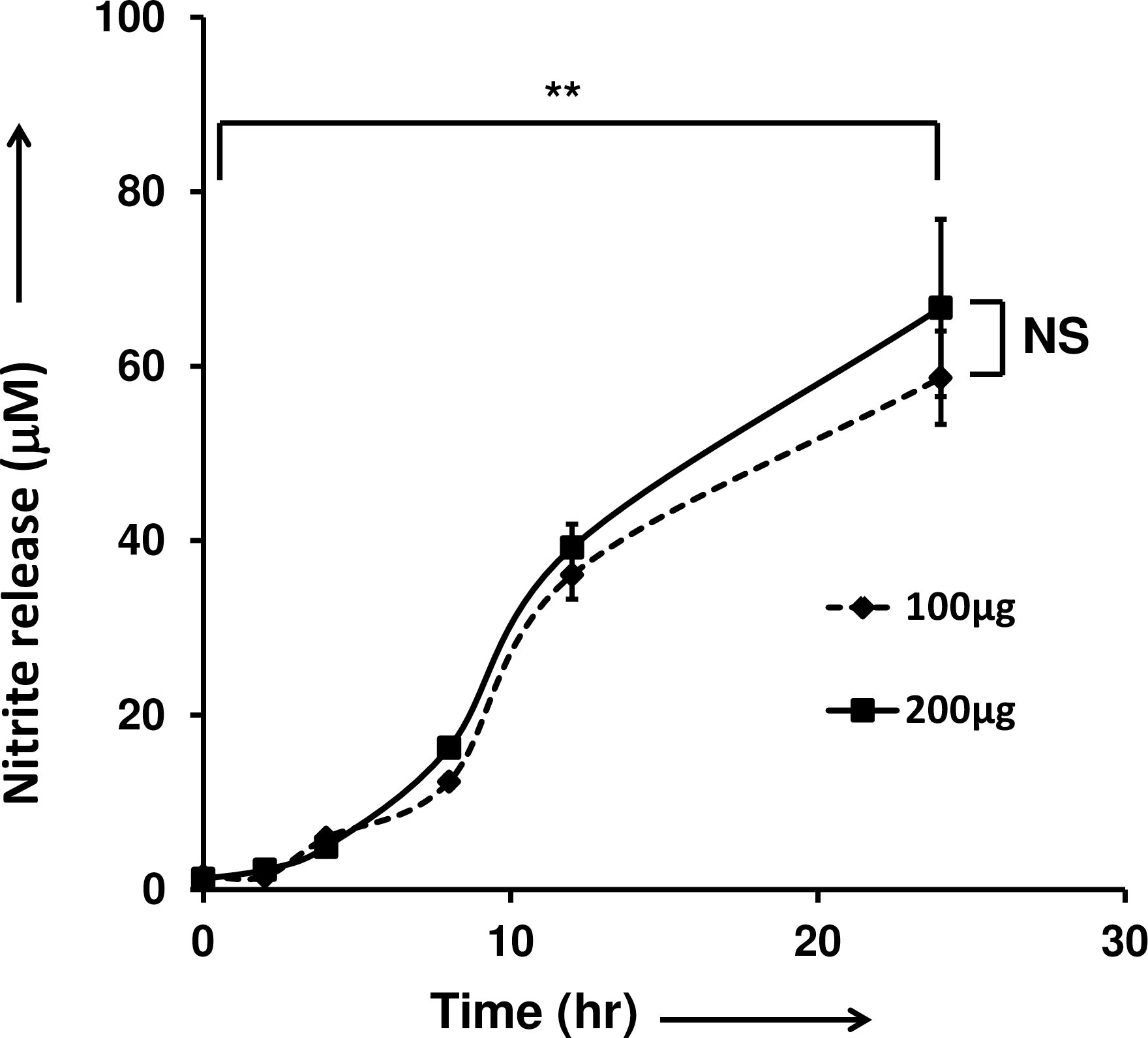

Supplement: S4 Fig — RAW 267.4 cells were cultured with 100 and 200μg/ml of soluble ICs for different time points (0–24 hr). The culture supernatant was collected at different time points, and the nitrite concentration was determined using the Griess reagent. The cells were harvested at the same time point and lysate was analyzed for iNOS expression (Fig 3 and S3 Fig). Data are average of three independent experiments. P<0.05 considered as significant (*) and P<0.005 as highly significant (**), NS: non-significant. (TIF) [file pone.0153620.s004.tif]
